# Supplementary material for: Myocardial Tissue Characterization in Patients with Hypertensive Crisis, Positive Troponin, and Unobstructed Coronary Arteries: A Cardiovascular Magnetic Resonance-Based Study
Source: Diagnostics (Basel). 2023 Sep 14;13(18):2943. doi: 10.3390/diagnostics13182943 (PMC10527803; doi:10.3390/diagnostics13182943)
Supplement: Supplementary file 1 [file diagnostics-13-02943-s001.zip › diagnostics-2546405-supplementary.pdf]

Table S1: Comparison of cardiac magnetic resonance imaging findings.

Variable	Troponin-negative controls (n=24)	Troponin-positive, unobstructed coronary arteries (n=19)	*p*
Indexed LV EDV (ml/m2)	65 (59 to 71)	85 (68 to 100)	<0.001
Indexed LV ESV (ml/m2)	22 (16 to 32)	36 (24 to 70)	0.005
LV ejection fraction (%)	63 ± 12	52 ± 16	0.019
Indexed LV mass(g/m2)	80 (67 to 103)	100 (79 to 133)	0.017
LV systolic dysfunction, n(%)	4(17)	10(53)	0.012
Maximum wall thickness (mm)	14 ± 2.4	14.5 ± 2	0.483
LVH, n (%)	13 (54)	14 (74)	0.221
Asymmetric LVH, n (%)	4 (17)	2 (11)	0.678
Indexed LA volume (ml/m2)	30 (28 to 36)	44(32 to 56)	<0.001
Global T2-w SI Ratio	1.4 ± 0.2	1.4 ± 0.1	0.551
Basal T2-w SI Ratio	1.4 ± 0.2	1.5 ± 0.2	0.661
Mid-ventricular T2-w SI Ratio	1.4 ± 0.2	1.5 ± 0.2	0.309
Apical T2-w SI Ratio	1.4 ± 0.2	1.4 ± 0.2	0.984
Global native T1 (ms)	1026 ± 39	1042 ± 40	0.198
Basal native T1 (ms)	1023 ± 39	1052 ± 38	0.105
Mid-ventricular native T1 (ms)	1024 ± 45	1042 ± 45	0.214
Apical native T1 (ms)	1019 ± 41	1032 ± 49	0.374
Global T2 relaxation time (ms)	47 ± 2	49 ± 2	0.004
Basal T2 relaxation time (ms)	47 ± 2	48 ± 2	0.029
Mid-ventricular T2 relaxation time (ms)	48 ± 2	50 ± 3	0.003
Apical T2 relaxation time (ms)	48 ± 2	49 ± 2	0.007
Global ECV (%)	23 ± 3	25 ± 4	0.047
Basal ECV (%)	22 ± 4	25 ± 5	0.066
Mid-ventricular ECV (%)	22 ± 3	25 ± 4	0.008
Apical ECV (%)	24 ± 6	25 ± 4	0.460
LGE present, n (%)	12/24 (50)	14/17 (82)	0.050
Infarct-pattern, n(%)	0(0)	5/17(29)	-
Non-ischaemic, n(%)	12/24 (50)	9/17(53)	0.445

P value for test of two proportions. hs cTnT, high-sensitive cardiac troponin T; NT-proBNP, N-terminal prohormone of brain natriuretic peptide; LV, left ventricular; LV EDV, left ventricular end diastolic volume; LV ESV, left ventricular end systolic volume; LVH, left ventricular hypertrophy; SI, signal intensity; T2-w, T2-weighted; ECV, extracellular volume; LGE, late gadolinium enhancement.

Table S2: Correlation of high-sensitive cardiac troponin T with imaging biomarkers among the group with troponin-positive unobstructed coronary arteries.

Variable	Spearman's correlation coefficient	*p*
Indexed left ventricular EDV, mL/m^2	-0.096	0.694
Indexed left ventricular mass, g/m^2	-0.295	0.221
Indexed left atrial volume, mL/m^2	0.030	0.904
Left ventricular ejection fraction, %	-0.029	0.906
T2-weighted signal intensity ratio	-0.149	0.541
Global T2 time, ms	-0.039	0.875
Global native T1 time, ms	-0.070	0.775
Global extracellular volume fraction, %	-0.229	0.402

EDV, end-diastolic volume.

Table S3: Comparison of imaging parameters in troponin-positive unobstructed coronary artery group and delta 20% with the troponin-negative controls.

Variable	Troponin-negative controls (n=24)	Troponin-positive, unobstructed coronary artery with delta 20% (n=15)	*p*
Age in years, Mean ± SD	47 ± 14	58 ± 11	0.015
hs cTnT (ng/L)	11 (6 to 11)	193 (57 to 282)	<0.001
NT-proBNP (ng/L)	77 (22 to 396)	279 (52 to 981)	0.101
Indexed LV EDV (ml/m2)	65 (59 to 71)	77 (67 to 100)	0.003
Indexed LV ESV (ml/m2)	22 (16 to 32)	36 (24 to 70)	0.006
LV ejection fraction (%)	63 ± 12	51 ± 17	0.030
Indexed LV mass(g/m2)	80 (67 to 103)	98 (79 to 128)	0.054
LV systolic dysfunction, n(%)	4 (17)	8 (53)	0.253
Maximum wall thickness (mm)	14 ± 2.4	15 ± 2.1	0.338
LVH, n (%)	13 (54)	11 (73)	0.348
Indexed LA volume (ml/m2)	30 (28 to 36)	44 (32 to 55)	<0.001
Global T2-w SI Ratio	1.4 ± 0.2	1.5 ± 0.1	0.399
Basal T2-w SI Ratio	1.4 ± 0.2	1.5 ± 0.2	0.383
Mid-ventricular T2-w SI Ratio	1.4 ± 0.2	1.5 ± 0.2	0.163
Apical T2-w SI Ratio	1.4 ± 0.2	1.4 ± 0.2	0.966
Global native T1 (ms)	1026 ± 39	1027 ± 37	0.352
Basal native T1 (ms)	1023 ± 39	1058 ± 39	0.191
Mid-ventricular native T1 (ms)	1024 ± 45	1034 ± 41	0.484
Apical native T1 (ms)	1019 ± 41	1029 ± 40	0.449
Global T2 (ms)	47 ± 2	49 ± 2	0.091
Basal T2 (ms)	47 ± 2	48 ± 2	0.062
Mid-ventricular T2 (ms)	48 ± 2	49 ± 3	0.086
Apical T2 (ms)	48 ± 2	49 ± 2	0.044
Global ECV (%)	23 ± 3	26 ± 4	0.086
Basal ECV (%)	22 ± 4	25 ± 5	0.174
Mid-ventricular ECV (%)	22 ± 3	26 ± 4	0.020
Apical ECV (%)	24 ± 6	25 ± 4	0.203
LGE present, n (%)	12/24 (50)	13/14 (93)	0.008
Infarct-pattern, n(%)	0/24(0)	5/14 (36)	0.002
Non-ischaemic, n(%)	12/24 (50)	8/14 (57)	0.681

SD, standard deviation; hs cTnT, high-sensitive cardiac troponin T; NT-proBNP, N-terminal prohormone of brain natriuretic peptide; LV, left ventricular; EDV, end diastolic volume; ESV, end systolic volume; LVH, left ventricular hypertrophy; LA, left atrial; T2-w, T2-weighted; SI, signal intensity; ECV, extracellular volume; LGE, late gadolinium enhancement.

Table S4: Comparison of imaging parameters and biomarkers among patients with troponin-positive unobstructed coronary artery based on pattern of late gadolinium enhancement.

Variable	Non-ischaemic LGE (n=9)	Infarct-pattern LGE (n=5)	*p*
Age in years, Mean ± SD	58 ± 12	51 ± 7	0.298
Male, n(%)	7 (78)	3 (60)	0.579
Creatinine (μmol/L)	105 (94 to 110)	95 (73 to 111)	0.518
LDH (U/L)	253 (211 to 298)	286 (213 to 387)	0.788
Haemoglobin (g/L)	16 ± 1.7	14 ± 2.8	0.190
hs cTnT (ng/L)	103 (21 to 211)	302 (103 to 510)	0.060
NT-proBNP (ng/L)	512 (71 to 1266)	113 (42 to 252)	0.171
Indexed LV EDV (ml/m2)	100 (72 to 133)	67 (66 to 74)	0.019
Indexed LV ESV (ml/m2)	54 (26 to 79)	23 (20 to 27)	0.029
LV ejection fraction (%)	49 ± 16	67 ± 6	0.029
Indexed LV mass(g/m2)	111 (87 to 140)	79 (70 to 101)	0.083
LV systolic dysfunction, n(%)	5(67)	0(0)	0.033
Maximum wall thickness (mm)	15 ± 2.4	13.5 ± 1.6	0.083
LVH, n (%)	7 (78)	3 (60)	0.579
Indexed LA volume (ml/m2)	54 (37 to 57)	39 (32 to 46)	0.147
Global T2-w SI Ratio	1.4 ± 0.2	1.4 ± 0.1	0.518
Basal T2-w SI Ratio	1.5 ± 0.2	1.4 ± 0.2	0.298
Mid-ventricular T2-w SI Ratio	1.5 ± 0.2	1.5 ± 0.2	0.699
Apical T2-w SI Ratio	1.4 ± 0.2	1.4 ± 0.2	0.797
Global native T1 (ms)	1045 ± 34	1048 ± 52	0.898
Basal native T1 (ms)	1052 ± 34	1067 ± 49	0.606
Mid-ventricular native T1 (ms)	1046 ± 39	1045 ± 56	0.898
Apical native T1 (ms)	1038 ± 35	1033 ± 61	0.898
Global T2 (ms)	48 ± 2	50 ± 2	0.298
Basal T2 (ms)	48 ± 2	49 ± 2	0.364
Mid-ventricular T2 (ms)	49 ± 1	51 ± 4	0.298
Apical T2 (ms)	49 ± 1	50 ± 3	0.147
Global ECV (%)*	25 ± 3	27 ± 6	0.381
Basal ECV (%)	25 ± 4	26 ± 8	1.000
Mid-ventricular ECV (%)	25 ± 3	27 ± 5	0.714
Apical ECV (%)	24 ± 2	28 ± 6	0.381

SD, standard deviation; LDH, lactate dehydrogenase; hs cTnT, high-sensitive cardiac troponin T; NT-proBNP, N-terminal prohormone of brain natriuretic peptide; LV, left ventricular; EDV, end diastolic volume; ESV, end systolic volume; LVH, left ventricular hypertrophy; LA, left atrial; T2-w, T2-weighted; SI, signal intensity; ECV, extracellular volume; LGE, late gadolinium enhancement.

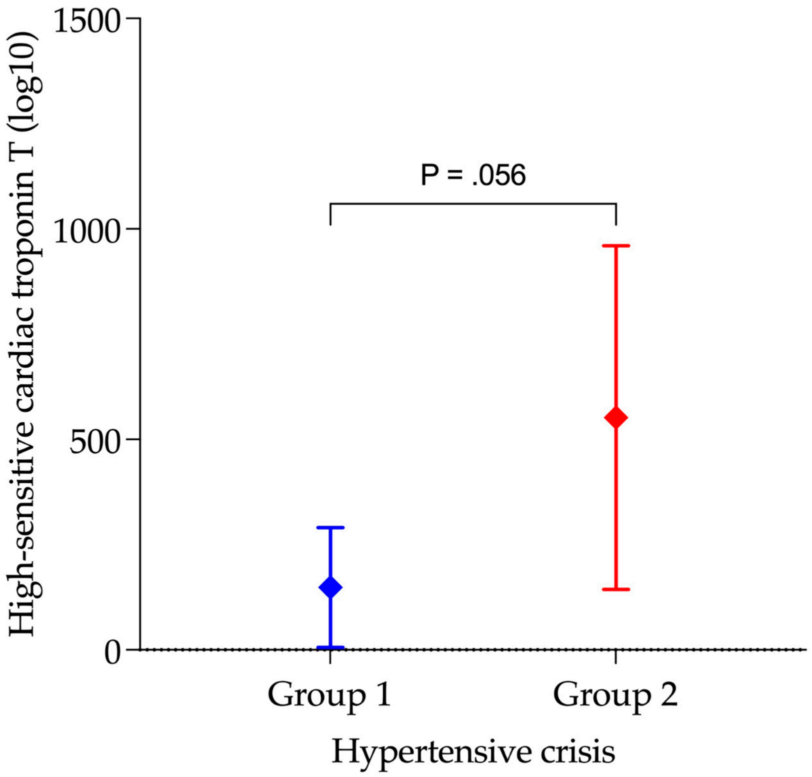

Figure S1: Comparison of cardiac troponin levels in patients with hypertensive crisis and troponin-positive unobstructed coronary arteries. Group 1, No infarct-pattern late gadolinium enhancement; Group 2, Infarct-pattern late gadolinium enhancement.
